# Determination

**Published:** 1874-10

**Authors:** 


### DETERMINATION.

A young student once placed his hands on the top rail of a fence, with the intention of jumping over, but he didn't do it. He was a young man of promise, but that was all ; he never made his mark in after life ; he went down to his grave, undistinguished among the vulgar millions. Very many years ago, an avalanche of rain came down upon the city, yet in five minutes the sun was shining brightly, while the water reached from curb to curb down a narrow street running off Broadway. Pedestrians were hurrying along to their business, or carelessly sauntered on the side walk, scarcely any two of a dozen or twenty acting alike. The first was a young man in plain clothing, who seemed to be walking on a wager, or as if it was indispensable to reach a certain point at a specified moment ; he came to the curb as if he had not noticed the angry little flood, but not turning his eye to the right or left, nor hesitating a single instant, he went straight across, the water coming up to his ankles ; nor did he stop an instant on the other side to adjust anything, nor observe if injury was done his clothing, and not looking behind, he passed rapidly on to his destination. The second was much like to him, until the instant of reaching the curb, when he suddenly halted, looked up and down, then across, shook his head, as much as to say, "I don't know about that," and crossed the street to the left, on the other side.

The third man also walked rapidly, but hearing the dash of the waters before he came in sight of them, he stopped short, turned round and went back. Another came after him and also halted, but not so quickly ; then he went a few steps further, halted again, looked down to his feet, and then to the water, as if to compare its depth to the height of his shoes, and studying a moment turned backward and walked slowly away.

The fifth man stopped a considerable distance before he reached the curb ; he looked troubled, did not seem to know whether he should go on or not; then he went a little further, stood still, started backward, and after a few steps halted, faced about, went a little further towards the little stream, looked wistfully across, and finally went home.

Another man came along carrying a heavy basket on his arm. He did not walk fast, but his gait was steady, and his tread firm; he was about forty-five, he had a wide mouth, and his lips were pressed together; he heard the running water, but did not alter his gait, nor did he when he came in full sight of the stream, nor even at the brink did he hesitate a moment, but stepped in, crossed to the other side and passed out of sight.

The reader can go back and study at his leisure the different characters, and will be able, with even a moderate amount of intelligence, to form a pretty good estimate of each one of the persons passing in review. The whole incident occurred within the time a person rings a door bell and a servant comes to open it; others came besides those mentioned, their conduct indicating varying shades of character, but not so distinctive. This occurred nearly forty years ago, but the mind has gone back to it multitudes of times as an illustration of the different characters one meets in the journey of life, and how that character may be read from the acts which flow from it. The reader may make use of this at any age, but especially if young, and should begin at once to train his mind to a wiser or more deliberate course of action. The first man did not count the cost, and plunged in regardless of consequences. He was unwilling to stop and think. Some young men do thus blunder into a fortune, or into success in life, but the multitude fail.

The last man had been schooled in the difficulties and sudden obstacles which meet all sooner or later; he thought as he went; and having concluded to take things as they come, the bad with the good, he deliberately and courageously pushed on. A wider and a more important use may be made of this narration by observing and judicious parents, in deciding from the little acts of their children what characteristics are in process of formation; what need cultivation, what need to be repressed, and what need to be modified. But in doing this never blame a child; either praise, or encourage, or lead. It is very likely that no human being, since Adam was created, has ever been made better by being found fault with, certainly not if in the presence of a third person; this is specially so with children, and if parents would only learn this lesson, an incalculable amount of "hurt feelings" would be prevented and many a child would be saved from a ruined life. There are very few more certain and speedy ways of driving a child into desperation than a persistent, everlasting way of finding fault, or making adverse criticisms on the part of parents; the uniform effect is to drive the child into recklessness, or bulldog obstinacy; then you can never do anything with him; he will be alike indifferent to entreaty, to scolding, to threats or punishment, and then he is lost.

Suppose you send your child for a book, by a description of the binding; he goes to the library for it, and comes back with a different one. That child is lacking in the valuable habit of attention; he did not notice your description of it with sufficient accuracy; this is the fault of three-fourths of the race. Do not scold him; describe it over again, and with the utmost patience and deliberation keep on sending him back until he brings the right one. But suppose he returns and says it is not there, if you are perfectly sure that it is there, then he lacks thoroughness, and unless this trait is nurtured the whole of after life will be embittered by a slip shod way of doing things, and pecuniary losses in every department of business will be an inevitable result, and always. Keep that child going back for that book if it takes a week; and show him that if he had at first gone over every shelf, book by book, he would have gotten it long ago. Be attentive, be thorough in all things.

